# Atypical Parkinsonism with Pathological Dopamine Transporter Imaging in Neuronal Ceroid Lipofuscinosis Type 5

**DOI:** 10.1002/mdc3.13562

**Published:** 2022-09-15

**Authors:** Lara M. Lange, Nathalie Schell, Sinem Tunc, Moneef Shoukier, Anne Weißbach, Yorck Hellenbroich, Norbert Brüggemann

**Affiliations:** ^1^ Institute of Neurogenetics University of Lübeck Lübeck Germany; ^2^ Department of Neurology University Hospital Schleswig‐Holstein Lübeck Germany; ^3^ Pediatrics Department University Hospital Essen Essen Germany; ^4^ Institute of Systems Motor Science, University of Lübeck Lübeck Germany; ^5^ Prenatal Medicine Munich, Department of Molecular Genetics Munich Germany; ^6^ Institute of Human Genetics, University of Lübeck Lübeck Germany; ^7^ Center for Brain, Behavior and Metabolism University of Lübeck Lübeck Germany

**Keywords:** neuronal ceroid lipofuscinosis, CLN5 disease, parkinsonism, genetic, imaging

Neuronal ceroid lipofuscinoses (NCLs) represent severe, commonly autosomal‐recessively inherited, progressive neurodegenerative diseases mainly affecting children and young adults.^1^ To date, there are at least 13 different genes, mutations of which cause various subtypes.^2^ NCLs commonly show a broad phenotypic spectrum, including continuous loss of vision, various movement disorders, progressive mental deterioration, and seizures. We here report on a patient with adult‐onset NCL type 5 with atypical parkinsonism and a pathological DaTSCAN partially responding to dopaminergic treatment.

## Case Report

**FIG. 1:**
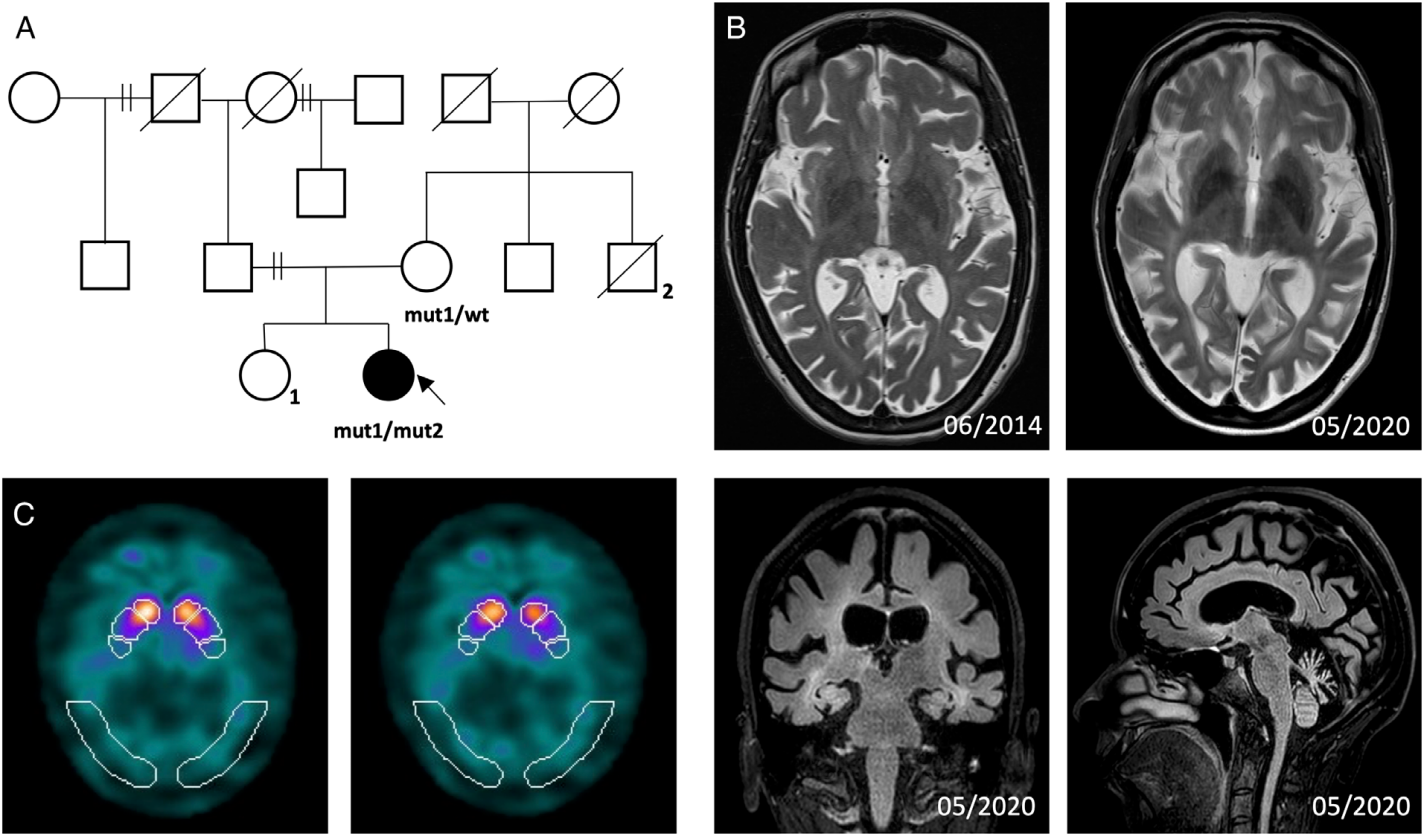
*Pedigree, DaTSCAN and MRI imaging of the index case*. (**A**) Pedigree of the family. The index case (arrow) carried both *CLN5* variants (mut1: C.486 + 4dupA and mut2: C.575A > G, p.Asn192Ser; NM 006493). The neurologically healthy mother carried one of these variants (mut1: C.486 + 4dupA). Other family members were not available for genetic testing.^1^ The sister suffered from multiple sclerosis and structural epilepsy.^2^ An uncle was reported to have suffered from the English disease. (B) MRI imaging reveals progressive total brain volume reduction and basal ganglia iron deposition, mostly pronounced in the parieto‐occipital region as well as progressive leukoencephalopathy. Wt, wildtype allele. (C) DaTSCAN of the index case (10/2020) shows an asymmetric reduction of presynaptic dopamine transporter density with a rostro‐caudal gradient.

A 35‐year‐old German woman presented with an 8‐year history of progressive speech and gait disturbance with recurrent falls. The neurological examination revealed cognitive dysfunction, ideomotor apraxia, saccadic pursuit, square wave jerks, gaze‐evoked nystagmus, hypokinetic‐rigid parkinsonism, lower limb spasticity, generalized dystonia, and cerebellar ataxia (see Video 1). Over the disease course, all symptoms were progressive with severe dysarthrophonia, parkinsonism, and rapidly progressive mental deterioration becoming most prominent. Additionally, there was urinary incontinence and continuous deterioration of visual acuity. The remaining medical history, including development and childhood history, was unremarkable. Apart from a sister with reported multiple sclerosis, there were no neurological diseases in the family (see Fig 1A). Unfortunately, the patient's sister was not available for a clinical or genetic evaluation.

**Video 1:** The patient presented with generalized dystonia affecting the limbs and trunk. While holding her arms in front of her chest there is dystonic posturing of both arms associated with myoclonic jerks. Further, there are signs of cerebellar ataxia (dysmetria) and parkinsonism (bradykinesia and decrement affecting both, upper and lower limbs). Independent standing up and walking is possible, but difficult and insecure. Her gait is slightly wide‐based, slow, and associated with dystonic posturing and reduced arm swing. The pull test revealed a slight postural instability.

Over the course of six years, cranial MRI revealed progressive brain atrophy, most pronounced in the cerebellum and parieto‐occipital lobes, and leukoencephalopathy (see Fig 1B). The examination of the CSF revealed isolated oligoclonal bands, which we interpreted as an incidental finding since there were no additional findings indicating a responsible cause, eg, no demyelinating lesions pointing towards multiple sclerosis. Spinal MRI, EEG, and nerve conduction studies were unremarkable. An ophthalmological examination showed distinct cone dystrophy with a severe bilateral reduction of visual acuity (right 0.1–1, left 0.05). The visually evoked cortical potentials were unremarkable, and the MRI revealed no signs of optic neuropathy, so we assume retinopathy as the cause of visual loss.

Based on the history, the clinical examination, and the performed diagnostics, several differential diagnoses were considered. The combination of various movement disorder phenotypes combined with progressive cognitive and visual impairment and the remarkable imaging made us suspect an adult‐onset neuronal ceroid lipofuscinosis (NCL), although the patient's symptoms would also fit into the spectrum of complicated hereditary spastic paraplegia (SPG), eg, SPG11 or SPG15. We initially screened palmitoyl‐protein thioesterase, tripeptide peptidase, and beta‐galactosidase levels which were normal, making NCL1 and 2 unlikely. Subsequent exome sequencing revealed two heterozygous variants in the *CLN5* gene (c.486 + 4dupA and c.575A > G, p.Asn192Ser). One variant was maternally inherited, the father was unavailable for testing. The missense variant has been reported before (https://www.ucl.ac.uk/ncl/CLN5mutationtable.htm), whereas the second mutation was novel and proven to cause aberrant splicing with exon skipping. Both variants were absent from genetic databases (https://gnomad.broadinstitute.org) and predicted to be disease causing by in‐silico prediction tools (*MutationTaster* and CADD score > 20 points). In keeping with the phenotypic presentation, we thus diagnosed an adult‐onset NCL type 5 (CLN5 disease).

Due to prominent parkinsonism, we initiated levodopa/benserazide (to date, 200/50 mg three times per day) replacement therapy, which resulted in a slight improvement of rigidity, bradykinesia, and psychomotor speed, and subjectively also general mobility. An additional DaTSCAN showed an asymmetric reduction of presynaptic dopamine transporter density with a rostro‐caudal gradient, a finding being characteristic for neurodegenerative parkinsonism (see Fig 1C).

## Discussion

*CLN5* deficiency causes a form of NCL, referred to as CLN5 disease usually with late‐infantile and rarely adulthood onset. Adult‐onset NCLs can be diagnostically challenging. They are rare, present with a broad phenotypic spectrum and the evaluation of pathological features may be difficult due to limited storage in accessible peripheral tissue and the additional accumulation of age‐related lipofuscin which could be misinterpreted as pathological.^3^ Despite genetic heterogeneity, the subgroups share histopathological and clinical characteristics. Common features shared by all NCLs include the degeneration of nerve cells mainly in the cerebral and cerebellar cortex and lysosomal accumulation of autofluorescent ceroid lipopigments leading to lysosomal dysfunction,^1^, ^2^ which classifies them as lysosomal storage disorders. Endo‐lysosomal dysfunction plays an important role in several neurodegenerative diseases, including NCLs and also parkinsonism. Thus, a mechanistic link between both entities does not seem too far‐fetched. Mutations in the “lysosomal” gene *ATP13A2* for example, alternatively known as the *CLN12* gene, not only cause CLN12 disease but also a rare form of autosomal recessive juvenile‐onset atypical parkinsonism (PARK‐*ATP13A2*, Kufor‐Rakeb syndrome)^4^ and complicated hereditary spastic paraplegia (SPG78).^5^ Interestingly, a recent study showed an up‐regulation of the autophagy‐related *alpha‐synuclein gene* (*SNCA*) in *CLN5* deficient cells.^6^ *SNCA* encodes for the α‐syn protein, which is well known for its role in the pathogenicity of Parkinson's disease (PD). However, whether this *SNCA* up‐regulation has a pathophysiological relevance in NCLs needs to be further elucidated.^6^ Furthermore, in addition to a presynaptic nigrostriatal deficit shown by a DaTSCAN in our patient, (123) I‐IBZM SPECT performed in patients with CLN4 showed loss of postsynaptic D2 receptor binding in the striatum.^7^ This indicates that both, presynaptic nigral cell loss and postsynaptic striatal degeneration plays a role in the pathogenesis of parkinsonism in NCLs.

Parkinsonism related to NCL has only been rarely reported. To our knowledge, this is the first case with atypical parkinsonism related to genetic variants in the *CLN5* gene. Response of parkinsonian features to levodopa therapy has previously been reported in patients with CLN2, CLN4, and CLN6.^7^, ^8^, ^9^ Additionally, some adult‐onset cases with *CLN5* variants and a similar phenotype to our patient, including speech and gait impairment, cerebellar ataxia, and visual impairment, have been described.^10^ Typical clinical findings of all NCLs are continuous loss of vision, various movement disorders, progressive mental deterioration, seizures, and eventually premature death,^11^ many of which are present in our patient.

The phenotypic presentation of our patient, notably in combination with a pathological DaTSCAN, points towards CLN5 disease as a rare differential diagnosis to early‐onset parkinsonism with atypical signs like dementia, dystonia, ataxia, and visual impairment; however, symptoms can still improve through dopaminergic therapy.

## Author Roles

(1) Research project: A. Conception, B. Organization, C. Execution; (2) Statistical Analysis: A. Design, B. Execution, C. Review and Critique; (3) Manuscript: A. Writing of the first draft, B. Review and Critique.

L.M.L.: 1B, 3A.

N.S.: 1B, 3B.

S.T.: 1B, 1C, 3B.

M.M.S.: 1C, 3B.

A.W.: 1B, 3B.

Y.H.: 1B, 3B.

N.B.: 1A, 1B, 3B.

## Disclosures

**Ethical Compliance Statement:** The study was approved by the Ethics Committee of the University of Luebeck. The patient gave written informed consent to participate in this study and for this study to be published in a scientific journal. We confirm that we have read the Journal's position on issues involved in ethical publication and affirm that this work is consistent with those guidelines.

**Funding Sources and Conflicts of Interest:** Dr. Brüggemann is funded by the DFG (BR4328.2–1, GRK1957) and the Michael J. Fox Foundation. Anne Weissbach receives funding from the Else Kröner‐Fresenius Foundation (EKFS, 2018_A55), the German Research Foundation (DFG, WE 5919/2–1, WE 5919/4–1, WE 5919/5–1) and the Dystonia Medical Research Foundation. She receives the Edmond J. Safra Movement Disorders Reaseach Career Develeopment Award from the Michael J. Fox Foundation. None of the contributing authors has any conflict of interest.

**Financial Disclosures for the Previous 12 Months:** Dr. Brüggemann received honaria from Abbott, Abbvie, Biogen, Biomarin, Bridgebio, Centogene and Zambon.

## References

[1] Jalanko A , Braulke T . Neuronal ceroid lipofuscinoses. Biochim Biophys Acta 2009;1793(4):697–709. 19084560 10.1016/j.bbamcr.2008.11.004

[2] Mukherjee AB , Appu AP , Sadhukhan T , Casey S , Mondal A , Zhang Z , Bagh MB . Emerging new roles of the lysosome and neuronal ceroid lipofuscinoses. Mol Neurodegener 2019;14(1):4. 30651094 10.1186/s13024-018-0300-6 PMC6335712

[3] Berkovic SF , Staropoli JF , Carpenter S , et al. Diagnosis and misdiagnosis of adult neuronal ceroid lipofuscinosis (Kufs disease). Neurology 2016;87(6):579–584. 27412140 10.1212/WNL.0000000000002943 PMC4977374

[4] Bras J , Verloes A , Schneider SA , Mole SE , Guerreiro RJ . Mutation of the parkinsonism gene ATP13A2 causes neuronal ceroid‐lipofuscinosis. Hum Mol Genet 2012;21(12):2646–2650. 22388936 10.1093/hmg/dds089 PMC3363329

[5] Estrada‐Cuzcano A , Martin S , Chamova T , et al. Loss‐of‐function mutations in the ATP13A2/PARK9 gene cause complicated hereditary spastic paraplegia (SPG78). Brain 2017;140(2):287–305. 28137957 10.1093/brain/aww307 PMC5278306

[6] Adams J , Feuerborn M , Molina JA , Wilden AR , Adhikari B , Budden T , Lee SY . Autophagy‐lysosome pathway alterations and alpha‐synuclein up‐regulation in the subtype of neuronal ceroid lipofuscinosis, CLN5 disease. Sci Rep 2019;9(1):151. 30655561 10.1038/s41598-018-36379-z PMC6336884

[7] Nijssen PC , Brusse E , Leyten AC , Martin JJ , Teepen JL , Roos RA . Autosomal dominant adult neuronal ceroid lipofuscinosis: Parkinsonism due to both striatal and nigral dysfunction. Mov Disord 2002;17(3):482–487. 12112194 10.1002/mds.10104

[8] Cherian A , PD K , Paramasivan NK , Krishnan S . Pearls & oy‐sters: Levodopa‐responsive adult NCL (type B Kufs disease) due to CLN6 mutation. Neurology 2021;96(21):e2662–e2665. 33875558 10.1212/WNL.0000000000011997

[9] Le NM , Parikh S . Late infantile neuronal ceroid lipofuscinosis and dopamine deficiency. J Child Neurol 2012;27(2):234–237. 21940688 10.1177/0883073811419261

[10] Mancini C , Nassani S , Guo Y , et al. Adult‐onset autosomal recessive ataxia associated with neuronal ceroid lipofuscinosis type 5 gene (CLN5) mutations. J Neurol 2015;262(1):173–178. 25359263 10.1007/s00415-014-7553-y

[11] Williams RE , Mole SE . New nomenclature and classification scheme for the neuronal ceroid lipofuscinoses. Neurology 2012;79(2):183–191. 22778232 10.1212/WNL.0b013e31825f0547

